# Analysis of the Antibacterial Properties of Compound Essential Oil and the Main Antibacterial Components of Unilateral Essential Oils

**DOI:** 10.3390/molecules28176304

**Published:** 2023-08-28

**Authors:** Anjiu Zhao, You Zhang, Feng Li, Lin Chen, Xingyan Huang

**Affiliations:** 1 College of Forestry, Sichuan Agricultural University, Chengdu 611130, China; anjiu_zhao@sicau.edu.cn (A.Z.); iszhangyoyo@163.com (Y.Z.); 2 Research Institute of Characteristic Flowers and Trees, School of Landscape Architecture, Chengdu Agricultural College, Chengdu 611130, China; li_feng2016@126.com

**Keywords:** essential oil, compound, bacteriostasis, chemical composition

## Abstract

Plant essential oils are widely used in food, medicine, cosmetics, and other fields because of their bacteriostatic properties and natural sources. However, the bacteriostatic range of unilateral essential oils is limited, and compound essential oil has become an effective way to improve the antibacterial properties of unilateral essential oils. In this study, based on the analysis of the antibacterial properties of Chinese cinnamon bark oil and oregano oil, the proportion and concentration of the compound essential oil were optimized and designed, and the antibacterial activity of the compound essential oil was studied. The results showed that the antibacterial activity of Chinese cinnamon bark oil was higher than that of oregano oil. The compound essential oil prepared by a 1:1 ratio of Chinese cinnamon bark oil and oregano oil with a concentration of 156.25 ppm showed an excellent antibacterial activity against *Escherichia coli* and *Staphylococcus aureus*. The GC-MS results showed that cinnamaldehyde was the main antibacterial component of Chinese cinnamon bark essential oil, and carvacrol and thymol in oregano oil were the main antibacterial components.

## 1. Introduction

The active components of natural biomass exist widely in nature and have the advantages of being rich resources, renewable, low cost, and easy to obtain [1]. As an active component of natural biomass, plant essential oils have been widely extracted from flowers, fruits, seeds, roots, stems, bark, and other parts of plants [2]. They protect plants from bacteria during plant growth [3]. Cinnamon is a medium-large tree of *Lauraceae*. The essential oil extracted from cinnamon has good antibacterial and antioxidant activities [4]. Oregano, also known as hyssop, is a plant of the genus oregano of *Labiatae*. The oregano oil has the advantages of antibacterial, antioxidation, and immunity enhancing properties, among others [5]. Moreover, natural plant essential oils are widely used in food, medical and health, cosmetics, and other fields and have broad application prospects and excellent application values.

In recent years, there has been a sharp increase in infectious diseases reported globally, especially those caused by food microbial contamination [6]. *Escherichia coli* and *Staphylococcus aureus* are the primary bacteria that cause human infection. The bacteriostatic properties of natural plant essential oils have attracted more and more attention. The essential oil has various components, which can play different bacteriostatic effects, making it difficult for pathogens to produce drug resistance. At the same time, plant essential oils come from plant tissues with low harm to the environment and human beings. They can effectively reduce environmental pollution and human health risks caused by synthetic antibiotics [7]. Bourrel et al. reported *Catnip* (*Nepeta cataria* L.) bacteriostatic properties of essential oil [8]. Thyme and peppermint essential oils have been found to have great potential as anti-tuberculosis drugs [9]. In addition, plant essential oils are also used as antibacterial agents in food [10]. The components of different essential oils are different and their antibacterial mechanisms may be different [11]. Some components in the essential oil act on the cell membrane, reducing the proportion of unsaturated fatty acids in the cell membrane, resulting in the loss of membrane fluidity, while some components can penetrate the cell membrane. It disrupts the osmotic balance of the cell membrane and causes the leakage of ions and other intracellular molecules, resulting in bacterial death [12]. Phenols in essential oils, for example, carvacrol, can improve the sensitivity of Gram-negative bacteria [13]. Alcohol inessential oils take the cell membrane as one of the action targets, and the balance of production and consumption of reactive oxygen species (ROS) is interrupted; oxidative stress then occurs, followed by cell damage, resulting in lipid peroxidation, cell membrane rupture, leakage of a large number of cell contents, and bacterial cell death [14]. Therefore, the bacteriostatic properties of natural plant essential oils enable them to replace some synthetic fungicides for their great application potential and market prospects [15].

The bacteriostatic and bactericidal activities of plant essential oils are related to their chemical type, concentration, and bacterial strains [16]. Usually, the bacteriostatic range of a single-component essential oil is limited, and a large dose is needed to achieve the inhibitory effect on low-sensitive pathogens [6]. The compound of essential oil is derived using the method of mixing single-component essential oils with different proportions and concentrations [17]. The minimum inhibitory concentration (MIC) of oregano and thyme compound essential oils was found to be significantly lower than that of unilateral essential oils [18]. The compound essential oil of citronella essential oil, cinnamon essential oil, and basil showed a synergistic effect on *Escherichia coli* and *Salmonella*, but an antagonistic effect on *Staphylococcus aureus* [19]. Because the composition of essential oil is very complex, the synergistic effect is often attributed to the strong combination of the main antibacterial active components of essential oil. However, it is difficult to analyze the specific reason for the antagonism. However, the bacteriostatic effects of compound essential oil based on Chinese cinnamon bark oil and oregano oil on *Escherichia coli* and *Staphylococcus aureus* have not been reported, and the differences and mechanisms of the two kinds of essential oils in terms of the bacteriostatic activity and mechanism are not clear.

In this study, Chinese cinnamon bark oil and oregano oil were used as research objects, and the antibacterial activity of unilateral essential oil was also studied. The concentration and proportion of compound essential oil were screened and optimized, and the antibacterial activity of compound essential oil on *Escherichia coli* and *Staphylococcus aureus* was studied. At the same time, the main components and bacteriostatic mechanisms of the essential oil were analyzed using GC-MS. The research content can provide a theoretical reference for the compounding and antibacterial application of Chinese cinnamon bark oil and oregano oil.

## 2. Results and Discussion

### 2.1. Bacteriostatic Activity of Unilateral Essential Oil

#### 2.1.1. Antimicrobial Sensitivity of Unilateral Essential Oil

**Figure 1:**
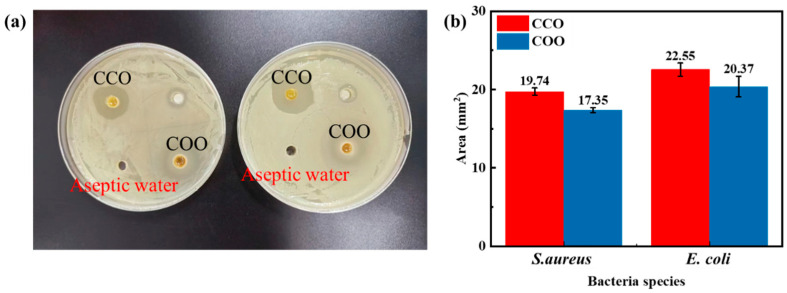
Antimicrobial sensitivity of unilateral essential oils. (**a**) The bacteriostatic zone of essential oil. (**b**) The area of bacteriostatic zone of essential oil. CCO: Chinese cinnamon bark oil; COO: Commercial oregano oil.

The bacteriostatic zone method is a mean of measuring the efficacy of bactericidal and antifungal agents [20]. The diameter of a bacteriostatic zone <10 mm indicates low sensitivity, 10–15 mm indicates medium sensitivity, and a bacteriostatic zone diameter >15 mm indicates high sensitivity [21]. Figure 1 shows the bacteriostatic zone morphology and area of Chinese cinnamon bark oil and oregano oil against *Escherichia coli* and *Staphylococcus aureus*. The bacteriostatic zone of the two kinds of essential oil was more significant than that of pure water (Figure 1a), and the bacteriostatic zone of the two tested bacteria was more than 15 mm. At the same time, the bacteriostatic zones of Chinese cinnamon bark oil against *Escherichia coli* and *Staphylococcus aureus* were more extensive than those of oregano oil (Figure 1b). The results showed that Chinese cinnamon bark oil and oregano oil had a highly sensitive antibacterial activity, and the bacteriostatic effect of Chinese cinnamon bark oil was slightly better than that of oregano oil.

#### 2.1.2. Effect of Unilateral Essential Oil Concentration on Bacteriostatic Activity

**Figure 2:**
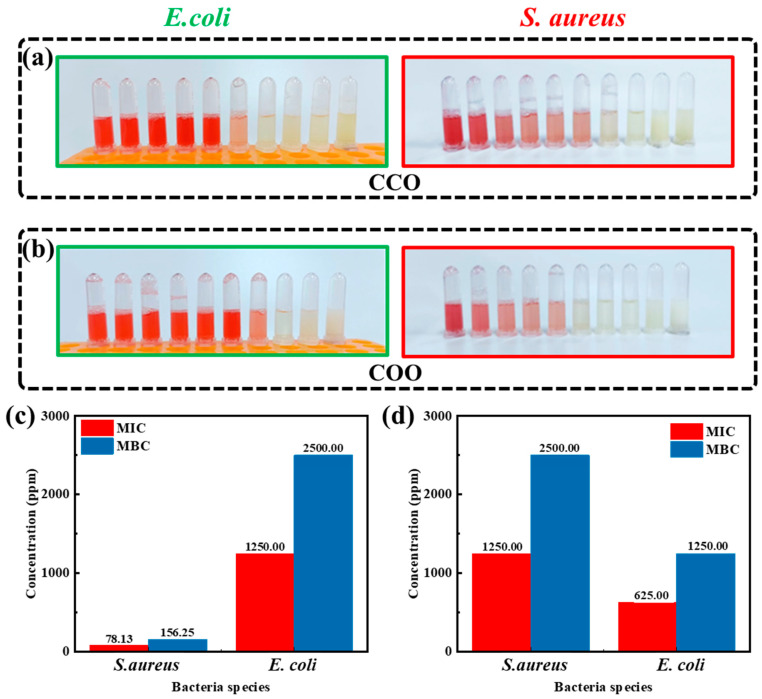
Effect of unilateral essential oil concentration on bacteriostatic activity. The color changes of different concentrations of Chinese cinnamon bark oil (**a**) and oregano oil (**b**) against *Escherichia coli* and *Staphylococcus aureus* by TTC staining. Minimum inhibitory concentration (MIC) and minimum bactericidal concentration (MBC) of Chinese cinnamon bark oil (**c**) and oregano oil (**d**) against *Escherichia coli* and *Staphylococcus aureus*.

After the bacteria were incubated with an ethanol solution of essential oil, the same amount of TTC solution was added to each tube, and the effect of different concentrations of essential oil–ethanol solution on bacterial activity was judged according to the color change trend. It can be seen from Figure 2a,b that both Chinese cinnamon bark oil and oregano oil had effects on the vitality of the two tested bacteria, and the color in the tube gradually changed from bright red to light pink, and then to no color, with the increase of the concentration of essential oil. When the concentration of essential oil ethanol solution was at the highest concentration gradient, the naked eye did not observe the color change. The results showed that determining bacterial activity by TTC staining could preliminarily determine the bacteriostatic substances and their effects on bacterial activity at different concentrations. The bacterial activity decreased gradually with the increase in the concentration of ethanol solution of essential oils, and both essential oils had concentration-dependent effects.

The bacterial activity gradually weakened under a high concentration of essential oil ethanol solution. Therefore, the centrifuge tube without color change was observed with the naked eye for the first time in each group. Theoretically, when the bacterial activity is very close to or lower than the detection limit of the TTC solution, the concentration of essential oil ethanol solution corresponding to the tube should be roughly relative to the MIC of essential oil. However, according to the literature study, the MIC of bacteriostatic substances obtained by the TTC staining method was relatively small, and further verification is needed to accurately determine the MIC value [22]. The MIC of Chinese cinnamon bark oil against *Escherichia coli* was 78.13 ppm, while that against *Staphylococcus aureus* was 1250.00 ppm. The MIC of oregano oil against the two tested bacteria was 1250.00 ppm and 625.00 ppm, respectively. Thus, it was demonstrated that the two essential oils have inhibitory effects on *Staphylococcus aureus* and *Escherichia coli*, and the inhibitory effect of Chinese cinnamon oil against *Escherichia coli* was more prominent. The results of MIC determination were consistent with the previous results of the bacteriostatic zone and bacterial activity of unilateral essential oil, and could provide a reference value for follow-up experiments.

### 2.2. Design and Antibacterial Activity of Compound Essential Oil

#### 2.2.1. Design of Proportion and Concentration of Compound Essential Oil

The design of compound essential oil from Chinese cinnamon bark oil and oregano oil is shown in Table 1. The numbers from 1 to 9 corresponded to nine combinations, and *E* and *S* represent *Escherichia coli* and *Staphylococcus aureus*, respectively. The numbers from 1 to 8 illustrate a series of high- to low-concentration gradients of compound essential oil. The compound essential oils of nine combinations were diluted according to the concentration gradient of the minimum inhibitory concentration of the unilateral essential oil, and the determined bacterial activity was used to narrow the range. After different combinations of essential oils were applied to the tested bacteria, the MIC of each group was preliminarily determined according to the results of bacterial activity staining. Then, the bacterial suspension was treated with this concentration to determine the bacteriostatic performance.

**Figure 3:**
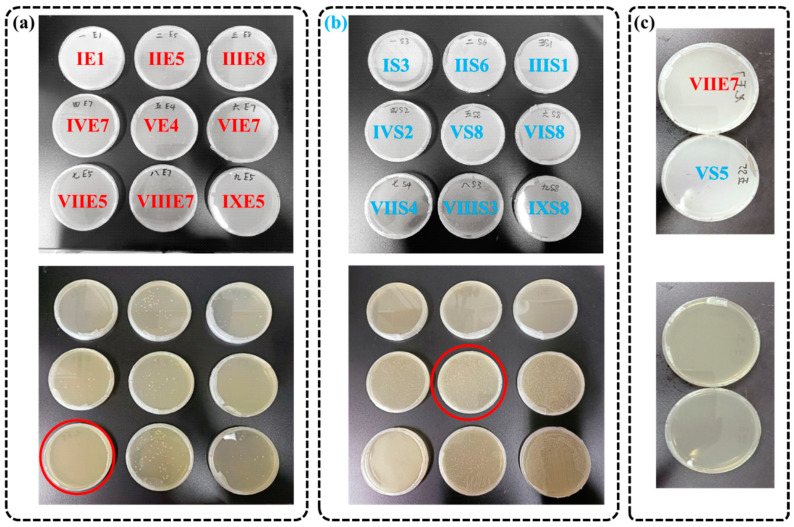
The bacteriostatic properties of essential oils with different concentrations and ratios. The growth status of *Escherichia coli* (**a**) and *Staphylococcus aureus* (**b**) under different concentrations and proportions of essential oils. (**c**) The growth status of *Escherichia coli* and *Staphylococcus aureus* under optimized essential oil ratio and concentration. Red circle means the best bacteriostatic property.

Figure 3a,b show the growth of bacteria at the MIC of each group according to the results of bacterial viability staining. According to the minimum inhibitory concentration and repeated experiments, the fifth concentration of combination VII was reduced to the seventh concentration, and it was found that the essential oil had the best bacteriostatic effect on *Escherichia coli*. When the eighth concentration of combination V increased to the fifth concentration, it was found that the essential oil had the best inhibitory effect on *Staphylococcus aureus* (Figure 3c). The minimum inhibitory concentration of the two essential oils against the two tested bacteria was lower than that of unilateral essential oils. At the same time, the fifth concentration gradient of combination V was higher than the minimum concentration that completely inhibited *Escherichia coli*, and it could also completely inhibit the growth of *Staphylococcus aureus*. Therefore, when the ratio of Chinese cinnamon oil to oregano oil was 1:1, and the concentration of 156.25 ppm, it was considered to have the best bacteriostatic effect on *Escherichia coli* and *Staphylococcus aureus*.

**Figure 4:**
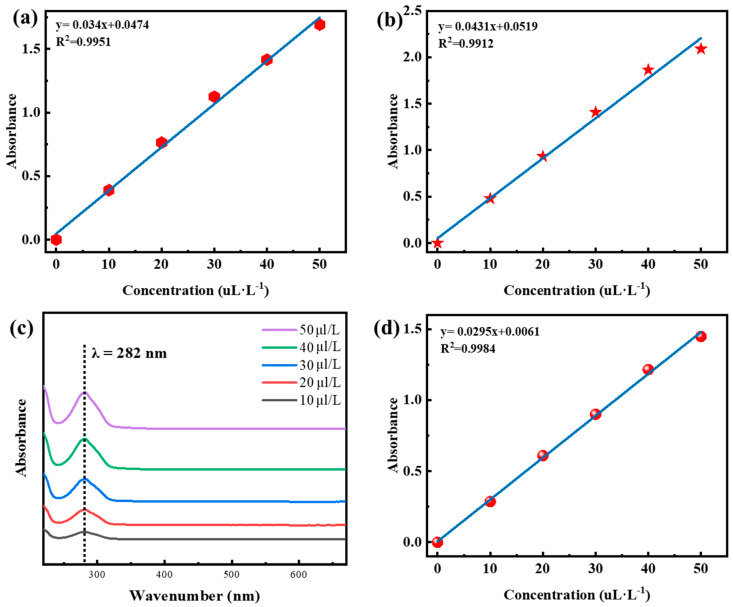
The concentration design of compound essential oil. The concentration standard curve of Chinese cinnamon bark oil (**a**) and oregano oil (**b**). (**c**) The absorbance characteristics of compound essential oils with different concentrations. (**d**) The standard curve of compound essential oil concentration. Hexagon means the UV-Vis absorbance of Chinese cinnamon bark oil; Five-pointed star represents the UV-Vis absorbance of oregano oil; Round is the UV-Vis absorbance of compound essential oils.

The concentration standard curves of Chinese cinnamon bark oil and oregano oil prepared via the volume dilution method using anhydrous ethanol as solvent are shown in Figure 4a,b. The maximum absorption wavelengths of Chinese cinnamon bark oil and oregano oil are 289 nm and 275 nm, respectively [23]. The standard curve of Chinese cinnamon oil obtained by ultraviolet spectrophotometry is y = 0.034x + 0.0474 and R^2^ = 0.9951. The standard curve of oregano oil is y = 0.0431x + 0.0519 and R^2^ = 0.9912. When Chinese cinnamon bark oil and oregano oil were mixed at 1:1 by volume, the maximum absorption wavelength of Chinese cinnamon bark oil and oregano oil at five concentrations was stable at 282 nm (Figure 4c). The standard curve of compound essential oil was y = 0.0295x + 0.0061 and R^2^ = 0.9984. The concentration design of essential oil could provide a reference by establishing the standard curve of essential oil.

#### 2.2.2. Bacteriostatic Property of Compound Essential Oil

**Figure 5:**
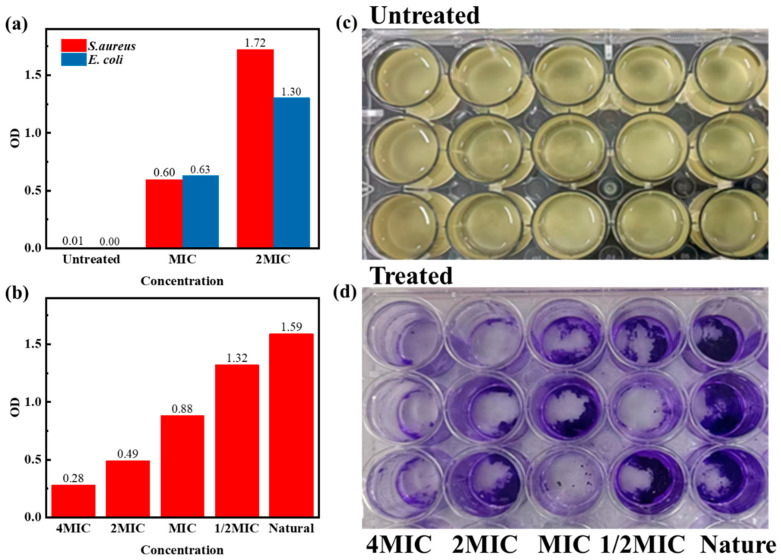
The bacteriostatic property of compound essential oil. (**a**) Effect of compound essential oil on protein leakage of tested bacteria. (**b**) Scavenging effect of different concentrations of compound essential oils on bacterial biofilm. Bacterial biofilm before (**c**) and after (**d**) treatment. OD means the absorbance of UV-Vis.

The antibacterial activity of the compound essential oil was evaluated by measuring the level of intracellular protein leakage and the clearance of bacterial biofilm [24]. It can be seen from Figure 5a that, compared with the untreated group, the absorbance of Chinese cinnamon bark oil and oregano oil mixed with oregano oil at 280 nm was significantly increased after the two kinds of test bacteria were treated for 24 h. The higher the absorbance value, the greater the leakage of active protein in bacterial suspension [25]. The protein leakage level of the two bacteria at the MIC (156.25 ppm) was the same. However, at 2MIC (312.50 ppm), the leakage level of *Escherichia coli* was higher than that of *Staphylococcus aureus*, which indicated that the compound essential oil had a more substantial destructive effect on *Escherichia coli* at high concentrations. Figure 5c shows the biofilm formed after two days of static culture of *Escherichia coli* and *Staphylococcus aureus*. Due to the continuous reproduction and expansion of bacteria, the culture medium in the culture plate gradually changed from clarification to turbidity, forming an aggregated colony above the liquid surface and forming a biofilm attached to the pore wall of the culture plate. Figure 5d shows the crystal violet staining results of the residual bacterial biofilm after the biofilm attached to the pore wall was treated with compound essential oil for 24 h. With the increase in essential oil concentration, the crystal violet staining of residual bacterial biofilm decreased. The crystal violet in each pore was extracted by anhydrous ethanol, and the absorbance value was determined. It was found that the absorbance value of crystal violet treated with the compound essential oil was lower than that of the untreated group (Figure 5b), which indicated that the compound essential oil could significantly remove the mature biofilm of the two kinds of test bacteria. In particular, the compound essential oil with 4MIC (625.00 ppm) had a severely destructive effect on the biofilm pre-formed by the two co-cultured bacteria.

### 2.3. Composition Analysis of Essential Oil

**Figure 6:**
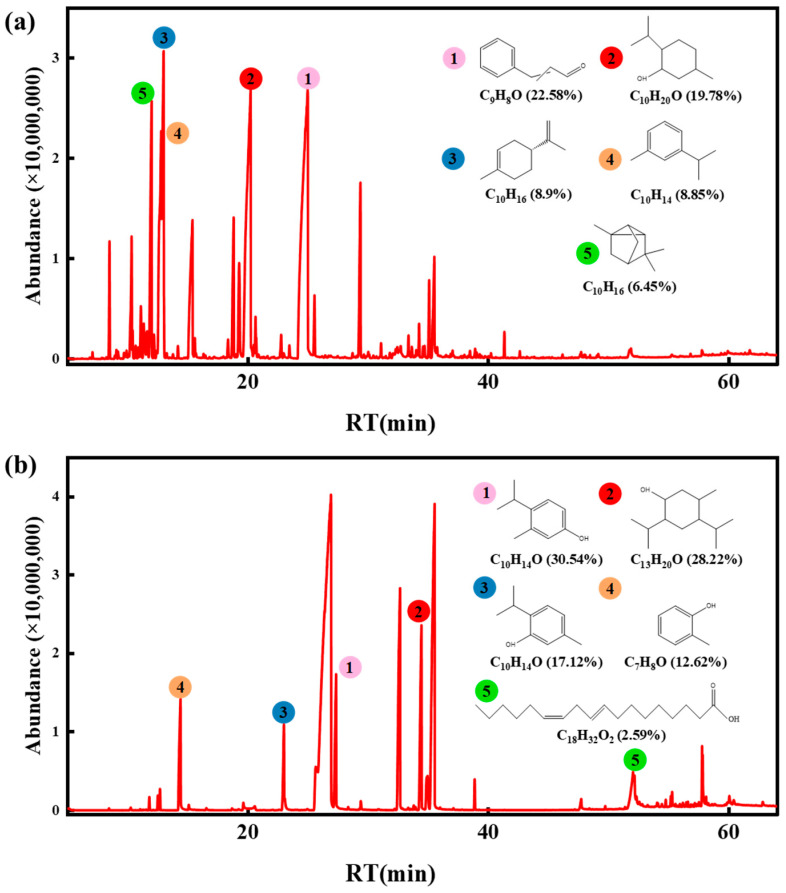
The total ion flow diagrams of essential oils. (**a**) Chinese cinnamon bark oil. (**b**) Oregano essential oil. RT represents retention time.

In Chinese cinnamon bark oil, twenty-eight compounds were identified using GC-MS (Figure 6a), including seven aldehydes, five esters, six alcohols, fifteen alkenes, one ketone, and six other compounds (Table S1). The five components with more relative contents were cinnamaldehyde (22.58%), (+)-isomenthol (19.78%), (+)-dipentene (8.90%), *m*-isopropyltoluene (8.85%), and tricyclo[2.2.1.0(2,6)]heptane,1,3,3-trimethyl (6.45%), which were consistent with the results of other related studies [26,27,28]. Cinnamaldehyde was the most abundant substance in cinnamon oil. Cinnamaldehyde can affect the distribution of fatty acids on the bacterial cell membranes, inhibit the activity of enzymes on the cell membrane, regulate the fluidity of cell membranes, enhance its permeability, inhibit the activity of intracellular ATP protease, affect cell energy supply, combine with intracellular proteins, hormones, and other factors, and affect cell normal division, thus producing the bacteriostatic effect [29]. Seventeen components were identified in oregano essential oil (Figure 6b), such as phenols, olefins, alcohols, and some esters (Table S2). The components with more relative contents were carvacrol (30.54%), 2,4-diisopropyl-5-methylphenol (28.22%), thymol (17.12%), *o*-cresol (12.62%), and linoleic acid (2.59%). Phenolic compounds in oregano essential oil can destroy the membrane structure by interfering with the bacterial cell wall’s outer membrane, release outer membrane lipopolysaccharides and embed in the phospholipid bilayer membrane [13], and prevent proteins from performing normal functions and chelating metal ions, thus achieving the bacteriostatic effect [30]. *Escherichia coli* is Gram-negative, while *Staphylococcus aureus* is Gram-positive. The peptidoglycan content in the Gram-negative bacteria’s cell wall is less than that of Gram-positive bacteria, and the cell wall of Gram-negative bacteria is thinner than that of Gram-positive bacteria. Cinnamaldehyde, carvacrol, and thymol in the essential oil mainly act on the bacteria’s cell wall [31]. Therefore, the inhibitory effect of essential oil on *Escherichia coli* was more evident than that on *Staphylococcus aureus.*

## 3. Materials and Methods

### 3.1. Materials

Commercial oregano oil (COO) and Chinese cinnamon bark oil (CCO) were purchased from Shanghai macklin Co., Ltd. (Shanghai, China). Gram-positive *Staphylococcus aureus* (*S. aureus*) and Gram-negative *Escherichia coli* (*E. coli*), numbered ATCC 29213 and ATCC 25922, were purchased from Beijing Biological Preservation Center (Beijing, China).

The bacterial suspension was prepared using the second-generation inclined bacteria of *Escherichia coli* (ATCC 25922) and *Staphylococcus aureus* (ATCC 29213). A ring of bacteria was taken from each of the one-time inoculation rings, and the plates were crossed on the sterile LB (Luria–Bertani) solid medium. The single colonies were cultured overnight in an incubator at 37 °C. The single colonies were selected and inoculated in 100 mL aseptic LB liquid medium and cultured overnight in a constant temperature shaker at 37 °C to activate the bacteria. An activated bacterial liquid was absorbed and added to a fresh LB liquid medium, continuing culture to the logarithmic growth phase. The bacterial suspension was diluted to 1 × 10^7^ CFU/mL to obtain the desired bacterial suspension.

### 3.2. Method

#### 3.2.1. Bacteriostatic Ability

##### Bacteriostatic Circle

Two kinds of bacterial suspensions with 100 μL concentration of 1 × 10^7^ CFU/mL were uniformly coated on the solid medium plate of aseptic LB. After the bacterial suspensions were wholly absorbed on the surface of the solid medium, a stopper borer with a diameter of 6 mm was used to drill three holes evenly in each plate, and 100 μL essential oil was added to each hole. The plate was sealed with a sealing film to prevent the essential oil from volatilization, and cultured in an incubator at 37 °C for 24 h. At the end of the culture, the diameter of the bacteriostatic circle was measured by an electronic Vernier caliper cross [32].

##### Detection of Bacterial Activity by TCC Method

Eight aseptic 10 mL centrifuge tubes were numbered to prepare a series of concentration gradient solutions of essential oil-anhydrous ethanol so that the concentration of essential oil in No. 1–8 tubes was 5000, 2500, 1250, 625, 312.5, 156.25, 78.125, and 39.0625 ppm. To the centrifuge tubes, different essential oil concentrations were added with 1 × 10^7^ CFU/mL bacterial suspension and essential oil-anhydrous ethanol gradient dilution solution, separately. After 24 h of culture, TTC (2,3,5-Triphenyltetrazolium chloride) staining solution was added to each tube so that the volume ratio of the three was 1:1:2. The blank control group was cultured with the same amount of LB liquid medium instead of bacterial suspension at 37 °C for 24 h at 220 rpm/min. The activity of bacteria in the centrifuge tube was judged according to the color of the centrifuge tube.

##### Minimum Inhibitory Concentration

The minimal inhibitory concentration (MIC) of essential oil against *Escherichia coli* and *Staphylococcus aureus* was determined using the double dilution method [33]. Eight aseptic 10 mL centrifuge tubes were numbered, and the concentration gradient solutions of essential oil-anhydrous ethanol were prepared according to Detection of Bacterial Activity by TCC Method. A 500 μL solution of essential oil was added to the aseptic centrifuge tube, and 500 μL bacterial suspension was added to each hole in the treatment group so that the final concentration of essential oil in the No. 1–8 tubes was 5000, 2500, 1250, 625, 312.5, 156.25, 78.125, and 39.0625 ppm. A quantity of 500 μL of broth medium instead of sample dilution was used as growth control, and 500 μL of broth medium instead of bacterial suspension was used as blank control. The samples were cultured in an incubator at 37 °C for 24 h, and 100 μL of bacterial suspension was smeared on a plate at the end of each tube. After 24 h, there was still no concentration of essential oil corresponding to the appearance of a colony, which was the minimum inhibitory concentration of essential oil.

##### Intracellular Protein Leakage

The two kinds of activated bacterial suspensions were washed with aseptic PBS buffer three times, then re-suspended in aseptic PBS buffer. The bacterial suspension concentration was adjusted to about 10^7^ CFU/mL. The final concentration of essential oil was the MIC and 2MIC of compound essential oil obtained by adding essential oil solution. Using sterile water as control, they were incubated in a constant temperature shaker at 37 °C for 24 h, centrifuged for 10 min at 8000 rpm/min, with 5 μL of supernatant. Then, they were added to a 96-well culture plate. A quantity of 200 μL of Coomassie brilliant blue staining solution was added to each well, gently shaken and mixed, and incubated at room temperature for 3 min. Finally, the absorbance at 595 nm was determined by a UV-Vis spectrophotometer, which was proportional to the leakage of intracellular active protein in the bacterial suspension.

##### Bacterial Biofilm Clearance

The two kinds of bacteria were co-cultured, and the concentration was adjusted to 1 × 10^7^ CFU/mL. The bacteria were added to a sterile 12-well plate and incubated in a constant temperature incubator at 37 °C for 24 h. After the completion of the culture, the bacteria that were not attached to the pore wall were carefully removed with sterile water, and fresh LB liquid medium and compound essential oil solution were then added to each hole, so that the final concentration of the essential oil solution was 1/2MIC, MIC, 2MIC, and 4MIC of the compound essential oil. Aseptic water instead of essential oil solution was used as the control group and cultured at 37 °C for 24 h. After the culture was completed, the culture plate was removed, and the cells were fixed with a formaldehyde solution. The fixed solution was discarded after 20 min. The bacterial biofilm was stained with 0.1% crystal violet solution for 30 min. After the staining was finished, it was washed with PBS buffer solution three times at room temperature to remove the excess staining solution. After that, the same amount of anhydrous ethanol was added to each hole for 15 min, and the absorbance of crystal violet solution in the pore was determined by a UV-Vis spectrophotometer at 595 nm. The residual amount of biofilm was proportional to the absorbance of crystal violet solution.

#### 3.2.2. GC-MS

Five times diluted oregano oil and undiluted Chinese cinnamon bark oil were dried with anhydrous sodium sulfate and filtered into a 1.5 mL injection bottle with a 0.22 μm disposable needle filter. Agilent 8890-5977BL1-2100 gas chromatography-mass spectrometry (Palo Alto, CA, USA) was used with an Agilent HP-5MS column (30 m × 0.25 mm × 0.25 μm). The injection port temperature was 250 °C, and the transmission line temperature was 280 °C. The split injection mode was adopted, and the shunt ratio was 20:1. The initial temperature was 50 °C, for 4 min, before heating to 110 °C with a 3 °C/min program, maintaining this for 2 min, heating to 210 °C with a 4 °C/min program, maintaining this for 2 min, heating to 280 °C with a 10 °C/min program, maintaining this for 4 min, and solvent delaying for 5 min.

Mass spectrometry conditions: ionization mode: EI; ion source temperature: 200 °C; quadrupole temperature: 150 °C; full scanning mode, scanning range: 35–550 aum. The components of the two kinds of commercial essential oils were analyzed with the standard mass spectra provided by NIST 2017 and related literature.

## 4. Conclusions

In this study, based on the analysis of the unilateral antibacterial activity of Chinese cinnamon bark oil and oregano oil, the proportion and concentration of compound essential oil were optimized and designed, the antibacterial activity of compound essential oil was studied, and the main chemical components of essential oil were analyzed. The results from examination of the bacteriostatic zone, bacterial activity, and minimum inhibitory concentration showed that the antibacterial activity of Chinese cinnamon peel oil was higher than that of oregano oil. After the design was optimized, the ratio and concentration of compound essential oil were found to be as follows: the ratio of Chinese cinnamon bark oil to oregano oil was 1:1 and the concentration was 156.25 ppm. The membrane integrities of *Escherichia coli* and *Staphylococcus aureus* were destroyed using a 1:1 compound essential oil of Chinese cinnamon bark oil and oregano oil at the concentration of 2MIC (312.50 ppm). At the concentration of 4MIC (62.5.00 ppm), the compound essential oil seriously damaged the biofilm pre-formed by two kinds of co-cultured bacteria and showed excellent bacteriostatic properties. GC-MS results showed that cinnamaldehyde was the main bacteriostatic component of Chinese cinnamon bark essential oil, and carvacrol and thymol in oregano essential oil were the main reasons for its bacteriostatic effect.

## Figures and Tables

**Table 1:** Design of proportion and concentration of compound essential oil. CCO: Chinese cinnamon bark oil; COO: Commercial oregano oil.

No	V_CCO_	V_COO_	Concentration Label	Concentration (ppm)	Bacteria Species
Ⅰ	1	9	8-1	39.0625-5000	*S. aureus*, *E. coli*
Ⅱ	2	8	8-1	39.0625-5000	*S. aureus*, *E. coli*
Ⅲ	3	7	8-1	39.0625-5000	*S. aureus*, *E. coli*
Ⅳ	4	6	8-1	39.0625-5000	*S. aureus*, *E. coli*
Ⅴ	5	5	8-1	39.0625-5000	*S. aureus*, *E. coli*
Ⅵ	6	4	8-1	39.0625-5000	*S. aureus*, *E. coli*
Ⅶ	7	3	8-1	39.0625-5000	*S. aureus*, *E. coli*
Ⅷ	8	2	8-1	39.0625-5000	*S. aureus*, *E. coli*
Ⅸ	9	1	8-1	39.0625-5000	*S. aureus*, *E. coli*

## Data Availability

Date is available as request to X.H.

## Supplementary Materials

The following supporting information can be downloaded at: https://www.mdpi.com/article/10.3390/molecules28176304/s1, Table S1: Chemical composition of Chinese cinnamon bark oil; Table S2: Chemical composition of oregano essential oil.

## Sample Availability

Sample is available as request to X.H.
