# Supplementary material for: Analysis of the Antibacterial Properties of Compound Essential Oil and the Main Antibacterial Components of Unilateral Essential Oils
Source: Molecules. 2023 Aug 28;28(17):6304. doi: 10.3390/molecules28176304 (PMC10489134; doi:10.3390/molecules28176304)
Supplement: Supplementary file 1 [file molecules-28-06304-s001.zip › molecules-2544418-supplementary.pdf]

**Table S1. Chemical composition of Chinese cinnamon bark oil.**

| No | Retention time | Molecular formula | Name of substance | Cas | Matching index | Content (%) |
|---|---|---|---|---|---|---|
| 1 | 24.916 | $C_9H_8O$ | Cyclohexanol,5-methyl-2-(1-methylethenyl)-, (1a,2a,5b)- | 104-55-2 | 99.6 | 22.58 |
| 2 | 18.369 | $C_{10}H_{18}O$ | Cyclohexanol,5-methyl-2-(1-methylethenyl)-, (1a,2a,5b)- | 29141-10-4 | 98.8 | 0.31 |
| 3 | 10.434 | $C_{10}H_{18}$ | cis-Pinane | 6876-13-7 | 96.0 | 0.28 |
| 4 | 15.604 | $C_{10}H_{16}$ | Bicyclo[4.1.0]hept-2-ene,3,7,7-trimethyl- | 554-61-0 | 95.4 | 0.24 |
| 5 | 33.365 | $C_{15}H_{24}$ | (-)-g-Cadinene | 39029-41-9 | 98.4 | 0.26 |
| 6 | 41.338 | $C_{16}H_{30}O_3$ | Tridecanoic acid,2-oxiranylmethyl ester; Tridecanoicacid, 2,3-epoxyprop | 63978-73-4 | 89.0 | 0.31 |
| 7 | 22.780 | $C_{10}H_{16}O$ | 5-methyl-2-(1-methylethylidene)-Cyclohexanone | 15932-80-6 | 97.4 | 0.41 |
| 8 | 12.216 | $C_{12}H_{20}O_2$ | 2-Cyclohexen-1-ol,3-methyl-6-(1-methylethyl)-, 1-acetate | 1204-30-4 | 92.1 | 0.34 |
| 9 | 11.363 | $C_{10}H_{18}$ | Cyclohexene,4-methyl-1-(1-methylethyl)- | 500-00-5 | 92.9 | 0.58 |
| 10 | 20.651 | $C_{10}H_{20}O$ | DL-Menthol | 89-78-1 | 99.1 | 0.56 |
| 11 | 34.250 | $C_{15}H_{24}$ | α-muurolene | 10208-80-7 | 99.2 | 0.46 |
| 12 | 11.469 | $C_8H_{18}O$ | 3-Octanol | 589-98-0 | 94.9 | 0.40 |
| 13 | 11.636 | $C_{10}H_{18}$ | cis-m-Menth-8-ene | 24399-15-3 | 83.7 | 0.63 |
| 14 | 35.086 | $C_{15}H_{24}$ | d-Cadinene | 483-76-1 | 96.9 | 1.13 |

| | | | | | | |
|---|---|---|---|---|---|---|
| 15 | 25.557 | $C_{12}H_{22}O_2$ | 5-methyl-2-(1-methylethyl) cyclohexanol acetate | 16409-45-3 | 99.0 | 0.93 |
| 16 | 11.122 | $C_{10}H_{16}$ | Myrcene | 123-35-3 | 95.4 | 0.87 |
| 17 | 24.986 | $C_9H_8O$ | trans-Cinnamaldehyde | 14371-10-9 | 94.8 | 4.47 |
| 18 | 19.292 | $C_{10}H_{18}O$ | Cyclohexanone,5-methyl-2-(1-methylethyl)-, (2R,5S)-rel- | 89-80-5 | 96.9 | 2.23 |
| 19 | 8.517 | $C_{10}H_{16}$ | alpha-Pinene | 80-56-8 | 98.6 | 1.41 |
| 20 | 35.515 | $C_{10}H_{10}O_2$ | 2'-Methoxycinnamaldehyde | 1504-74-1 | 99.1 | 2.51 |
| 21 | 29.374 | $C_{15}H_{24}$ | Tricyclo[4.4.0.02,7]dec-3-ene,1,3-dimethyl-8-(1-methylethyl)-, (1R,2S,6S,7S,8S)- | 3856-25-5 | 98.2 | 3.67 |
| 22 | 18.834 | $C_{10}H_{18}O$ | 5-methyl-2-(1-methylethyl)-, (2S-trans)-Cyclohexanone | 14073-97-3 | 98.7 | 2.97 |
| 23 | 13.016 | $C_{10}H_{16}$ | Cyclohexene,1-methyl-4-(1-methylethenyl)-, (4R)- | 5989-27-5 | 96.1 | 8.90 |
| 24 | 10.358 | $C_{10}H_{16}$ | beta-Pinene | 127-91-3 | 97.4 | 2.40 |
| 25 | 12.016 | $C_{10}H_{16}$ | Tricyclo[2.2.1.02,6]heptane,1,3,3-trimethyl- | 488-97-1 | 97.5 | 6.45 |
| 26 | 15.416 | $C_7H_{12}O4$ | Diethyl malonate | 105-53-3 | 98.5 | 6.07 |
| 27 | 20.251 | $C_{10}H_{20}O$ | DL-Menthol | 1490-04-6 | 99.1 | 19.78 |
| 28 | 12.810 | $C_{10}H_{14}$ | 1-methyl-3-(1-methylethyl)-benzen | 535-77-3 | 91.3 | 8.85 |

**Table S2. Chemical composition of oregano essential oil.**

| No | Retention time | Molecular formula | Name of substance | Cas | Matching index | Content (%) |
|---|---|---|---|---|---|---|
| 1 | 11.826 | $C_{10}H_{16}$ | Tricyclo[2.2.1.02,6]heptane,1,3,3-trimethyl- | 488-97-1 | 99 | 0.61 |
| 2 | 12.532 | $C_{10}H_{14}$ | 1-isopropyl-2-methylbenzene | 527-84-4 | 99 | 1.25 |
| 3 | 12.708 | $C_{10}H_{16}$ | Cyclohexene,1-methyl-5-(1-methylethenyl)-, (5R)- | 1461-27-4 | 97 | 0.74 |
| 4 | 14.419 | $C_7H_8O$ | o-Cresol | 95-48-7 | 99 | 12.62 |
| 5 | 19.643 | $C_{10}H_{20}O$ | Menthol | 15356-70-4 | 99 | 0.30 |
| 6 | 23.011 | $C_{10}H_{14}O$ | Thymol | 89-83-8 | 96 | 17.12 |
| 7 | 23.086 | $C_9H_{12}O$ | 3-(1-methylethyl)-Phenol | 618-45-1 | 88 | 1.41 |
| 8 | 27.350 | $C_{10}H_{14}O$ | o-Cymen-5-ol | 3228-02-2 | 97 | 30.54 |
| 9 | 29.406 | $C_{11}H_{16}O$ | Benzene,1-methoxy-4-methyl-2-(1-methylethyl)- | 31574-44-4 | 93 | 0.85 |
| 10 | 34.444 | $C_{13}H_{20}O$ | 2,4-Diisopropyl-5-methylphenol | 40625-96-5 | 97 | 28.22 |
| 11 | 38.867 | $C_{16}H_{26}O$ | 2,6-Ditert-butyl-4-ethylphenol | 4130-42-1 | 91 | 1.70 |
| 12 | 47.729 | $C_{16}H_{32}O_2$ | Palmitic acid | 57-10-3 | 99 | 0.41 |
| 13 | 52.031 | $C_{18}H_{32}O_2$ | Conjugated linoleic acid | 2420-56-6 | 84 | 2.59 |
| 14 | 55.283 | $C_{19}H_{36}O_3$ | Hexadecanoic acid,2-oxiranylmethyl ester | 7501-44-2 | 96 | 0.23 |

| | | | | | | |
|---|---|---|---|---|---|---|
| 15 | 57.771 | $C_{21}H_{38}O_4$ | 9,12-Octadecadienoicacid (9Z,12Z)-, 2-hydroxy-1-(hydroxymethyl)ethyl ester | 3443-82-1 | 84 | 0.68 |
| 16 | 57.825 | $C_{21}H_{38}O_3$ | 9-Octadecenoic acid(9Z)-, 2-oxiranylmethyl ester | 5431-33-4 | 95 | 0.58 |
| 17 | 60.037 | $C_{21}H_{38}O_4$ | 9,12-Octadecadienoicacid (9Z,12Z)-,2-hydroxy-1-(hydroxymethyl)ethyl ester | 3443-82-1 | 91 | 0.16 |
